# Increasing Access to Mental Health Services: Videogame Players’ Perspectives

**DOI:** 10.3390/ijerph20064772

**Published:** 2023-03-08

**Authors:** Emma L. van der Schyff, Rowena Forsyth, Krestina L. Amon, Brad Ridout, Andrew J. Campbell

**Affiliations:** Cyberpsychology Research Group, Biomedical Informatics and Digital Health Theme, School of Medical Sciences, Faculty of Medicine and Health, The University of Sydney, Sydney, NSW 2006, Australia

**Keywords:** videogaming, mental health, mental health services, gaming, help-seeking behavior

## Abstract

Young men’s mental health is at the forefront of global public health concerns. Young males, who have a high incidence of mental health disorders, are a population that accesses services at lower rates than females and makes up the majority of videogame players. By considering the unique perspectives of digitally connected individuals on mental health service delivery, interventions may be designed to address their needs with a higher likelihood of success. This study investigated international male videogamers’ perspectives on how their access to mental health services could be improved via an open-ended survey question. From a total of 2515 completed surveys, 761 responded to the qualitative question. Of these, the 71 responses that discussed access to and provision of mental healthcare services are reported in this article. Results suggest that digital mental health services were a promising way to reach this group. Anonymity and confidentiality were found to be important factors when considering online mental health services. Male videogame players identified a preference for both online and in-person services that are delivered synchronously, one-on-one with an expert practitioner, and readily available in settings that individuals find comfortable.

## 1. Introduction

The mental health of men, particularly young men, is a concern at the forefront of global public health. While women are more likely to be diagnosed with depression, suicide rates worldwide are higher among men [1]. Affleck, Carmichael and Whitley [2] note that mental health conditions among men are underdiagnosed due to reporting bias. This may be due to the fact that men have been found to underreport possible symptoms of mood disorders. Research also suggests that men are also significantly less likely to seek professional support for mental health [3]. Social norms associated with masculinity, such as stoicism and self-reliance, are one explanation for this gap [4]. To address this reluctance to seek help, clinicians need to consider the unique perspectives of men who experience mental ill-health and help-seeking in order to design services that better meet their needs and preferences. By incorporating these perspectives into the design and development process, patient outcomes can be maximized [5].

Understanding how and why individuals access support for their mental health and how to direct interventions to increase help-seeking behavior. Research suggests that early engagement with appropriate mental health services has the potential to increase quality of life and reduce the burden of disease [6]. In order to increase the rates of young adults seeking help, clinicians and researchers need to better understand why these individuals seek help in certain contexts. It is important to look at the population not as homogeneous but as sub-groups of individuals with shared interests to gain a rich understanding of their perspectives and experiences. By doing so, targeted interventions can be developed that address the specific needs of the group.

International government public health responses to the COVID-19 pandemic included requirements for individuals to physically distance themselves from others by staying indoors [7,8]. During this period, there was an increased demand for online mental health services [9]. Accessing services through online platforms may be particularly suitable for men, a group already identified as less likely to access mental health support than their female counterparts prior to the pandemic. While stigma still plays a part in the reason men do not seek support, [10] being able to access support from the comfort of home may appeal to this group due to it being more flexible and less intimidating. It is critical that online services and interventions are developed with the preferences of this group in mind. Videogame players are predominantly young men who engage with others online and have unique perspectives on online mental health service delivery.

It is estimated that there are over 3 billion videogame players worldwide [11]. Men make up the majority of videogamers. Estimates suggest around 55% of U.S. videogamers are male, according to 2021 figures [12]. Further, it is predicted that video gaming is the fastest-growing entertainment industry in the world [13]. Gaming is also an increasingly social pastime, where players can engage with others through multi-player game features. According to research done by the Entertainment Software Association [13], young adults (aged between 18 and 25 years old) are reported to value games for comfort and connection as opposed to older adults (aged 65+) who value games for passing time and mentally improving themselves. This engagement has spurred researchers and clinicians to develop serious videogames, which are videogames designed for a purpose other than entertainment. Two examples of this in the context of health are games developed for behavior change [14] and education [15]. While results suggest that serious videogames have shown promising results for their intended purpose [16,17] there are significant limitations to the development and maintenance of these types of games. These games face significant challenges to compete with commercial, off-the-shelf games developed by gaming companies with significant financial investment. Likewise, for sustained positive impact, serious games need to continuously develop and adapt to remain relevant to their audience. Investigating the effects of commercial videogames and the opinions and perspectives of their players is an important avenue for research.

Strong immersion in videogames has led scholars to use the term ‘escapism’ to describe when players seek distraction and relief from the unpleasantness of real life by participating in video gaming [18]. This engagement can be a diversion from real life for the player and has been associated with negative and positive behavioral outcomes. Some examples of the negative outcomes associated with escapism are depression [19], stress [20], and social withdrawal [21], while positive outcomes include achievement [22] and social connection [23]. For gamers, escaping from real life might look like reduced engagement with the person’s in-person support network and a reduced likelihood of seeking professional mental health services. Playing videogames has been shown to have some potential negative effects on those who participate in them. The DSM-5-TR notes that the effects of gaming and associated “neglect of other activities” are a condition warranting further research [24]. Withdrawal and reduced engagement in daily life, a phenomenon that research suggests may be as a result of videogaming [25], may lead to reduced social support and a reduced engagement with mental health services [26].

This study aimed to investigate how male videogame players could be encouraged to access mental health services. The study sought an international sample of current male videogamers due to games being digital spaces where individuals engage across geographic boundaries. Their perspectives were considered to draw conclusions about the preferences of this group for mental health service delivery.

## 2. Materials and Methods

Ethics approval was attained from the University of Sydney Human Research Ethics Committee (approval number: 2020/419). This included a commitment to the survey being confidential and anonymous (no personally identifiable information was collected in the survey).

Recruitment for the survey was done through a Twitter post (a Tweet) made by an Australian celebrity gamer, which advertised the voluntary, anonymous, and unincentivized online survey and included a link to complete it. At the time of tweeting this link, the gaming celebrity had over 1.8 million followers on the platform. This celebrity gamer was not paid or incentivized to promote the research. The survey was live between the 6 and 8 October 2021.

Upon clicking the link, participants were provided with a Participant Information Statement and asked to consent to the study by proceeding to answer the online survey questions presented using REDCap software. Individuals were eligible to participate in the study if they were male, over the age of 18, and self-identified as a videogame player. A videogame player was defined in the study as someone who actively engages with videogames (either by computer, console, or phone) at least once per month.

The survey collected quantitative and qualitative data. The quantitative component of the survey collected data on hours gamed, self-esteem, and social capital as they relate to help-seeking efficacy. At the end of the survey, the participants were asked “Is there anything that you think would better support you to access mental health services?” with the responses recorded as qualitative text-based data. From a total of 2515 completed surveys, 761 responded to the qualitative question. This study reports the findings of the qualitative component of the survey.

Some responses were ambiguous in nature and did not give enough detail on an aspect of online service delivery. Words such as “better”, “easier” and “more” were used, but it was unclear to what element of the help-seeking process the participant was referring without making assumptions. As such, some participant perspectives were excluded from the analysis. This is discussed in more depth in the limitations and avenues for future research section of the paper.

### Analysis Strategy

Thematic analysis was carried out on all the responses by authors EvdS and RF according to the four-phase procedure of (1) immersion in the data, (2) development of codes, and (3) refining these codes into categories and (4) combining these into themes [27]. This was done by hand and themes were agreed upon by both researchers, EvdS and RF. Following detailed reading and re-reading of the responses, the researchers grouped all 761 responses into 25 specific themes, and then these were merged as appropriate. These themes included codes such as ‘poor experiences with services’, ‘knowledge of services’, and ‘anonymity and confidentiality’.

This publication presents analysis of the 71 responses coded under the broad theme of ‘access to mental health services’ and encapsulates the following codes: ‘support in community centres’, ‘home, school, and university-based services’, ‘online services’, ‘in-person services’, ‘the consequences of not having access to mental health services’ and ‘anonymity and confidentiality’. These codes form the basis of the results reported.

## 3. Results

The mean age of the respondents to the qualitative component of the study was 21.75 (compared to 21.37 years for the participants who completed the full survey) with a standard deviation of 5.83. Most of the participants were based in North America (45.8%), followed by Europe (23.8%) and Australasia (17.4%) (there was no significant difference between this demographic breakdown and the wider cohort who completed the survey). More than half of the participants (52.6%) reported gaming every day, with 95.7% of the sample reporting gaming most days of the week.

**Figure 1:**
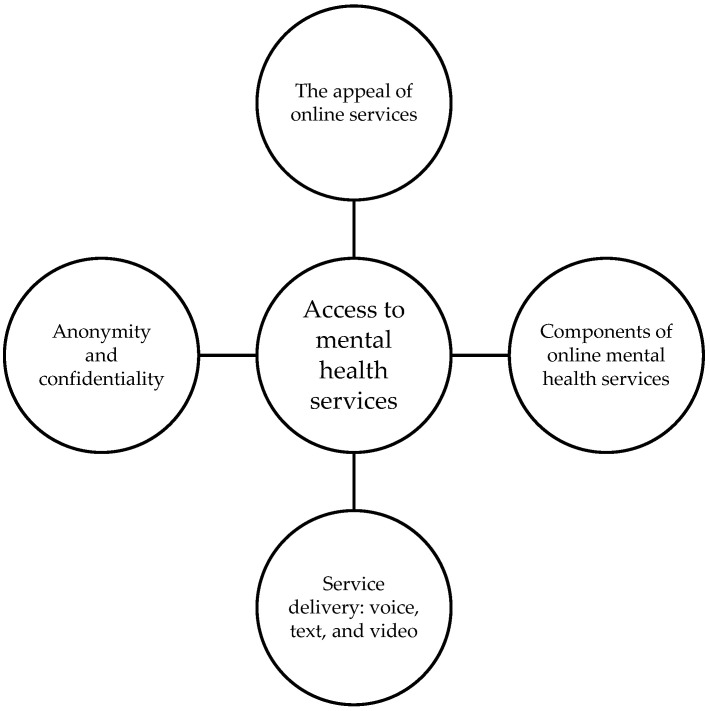
Four aspects of ‘Access to mental health services’.

The 71 responses reflected not only current experiences of accessing services but also how services could be better delivered using digital technologies. The themes were organized into ‘the appeal of online services’, ‘components of online mental health services’, ‘service delivery: voice, text, and video’ and ‘anonymity and confidentiality’ (see Figure 1).

### 3.1. The Appeal of Online Services

Participants noted the components of online mental health services that appealed to them. First, online help-seeking was viewed largely as being both comfortable and easy to access, with some participants noting that they viewed them more positively than traditional in-person services.

*“People might not feel comfortable actually going somewhere to get the help.”*

*“online services where I can talk with someone would help me open up more I feel.”*

*“I think online mental health services would be so much better than the traditional ones. As they are able to access to them from their house, and be more comfortable talking about it”*

*“online services where I can talk with someone would help me open up more I feel”*

The anxiety of attending in-person consultations was captured in some of the responses. ‘Being comfortable’ was noted by several participants as a facilitator of effective mental health treatment. The ability to access mental health services from the comfort of one’s own space was particularly appealing to the participants. Online help-seeking was viewed as comfortable and easy to access, providing individuals with a sense of control and autonomy over their own mental health care.

Further, they noted that booking a consultation and attending the appointment in person took a lot of courage:

*“Not everyone has the courage to ring or walk into a mental health service.”*

*“I don’t want to leave my home as I feel judged”*

*“most people with mental health have anxiety and don’t want to speak to a person in person or over the phone”*

For our participants, booking a consultation and attending the appointment in person may be particularly daunting, requiring a significant amount of courage and vulnerability from individuals. Participants noted that they may feel uncomfortable discussing personal or sensitive issues face-to-face with a mental health provider, especially if they have never sought mental health support before. The participants stated that online services enabled them to engage with services from home or a place that is comfortable for them. Participants reported greater comfort for online help-seeking and appointment-making experiences compared to in-person appointments.

### 3.2. Components of Online Mental Health Services

Participants discussed the features of mental health services that helped with accessing them online. One prominent feature of online services that appealed to participants is the potential for timely help-seeking interactions:

*“People don’t feel comfortable… having to book an appointment and wait[ing]. Some people need help right then and there.”*

*“the waiting times can cause a lot of problems”*

*“Less time spent waiting”*

*“An interim online service during the wait period”*

The appeal of online services was their ability to increase access to services by decreasing waiting times for appointments. Long wait times were noted as a significant barrier to our participants accessing mental healthcare.

Automated response-based services were raised as a possible mode of delivery, but participants noted their preference for this not to be at the expense of human-to-human interactions:

*“[online services where you can] actually be with people, rather than AI [Artificial Intelligence], as its more easier [sic] for a person to understand each other.”*

Another way participants saw the benefits of online services is through increased access to their own information:

*“The information… should be compiled online for everyone to see.”*

*“Information provision [of my own information]”*

Making it easier for clinicians and individuals to access mental health records was seen as a benefit of online mental health services. Access to mental health records enables individuals to better understand their mental health history, identify patterns, and make informed decisions about treatment options. It may have the additional benefit of enabling them to communicate more effectively with healthcare providers.

### 3.3. Service Delivery: Voice, Text, and Video

Some participants discussed how online services could be delivered. These tended to be idealized future versions of services rather than those that already existed. Various modalities for service delivery were discussed, including text, voice, and virtual reality. Several participants noted that they would prefer messaging services. This was noted as a promising way of connecting to clinicians, consistent with participants’ way of interacting with friends:

*“Typing your responses and taking the time to form your thoughts in your head instead of face to face with someone or over call where you have to be verbal and others around might be able to hear. I text my friends when I’ve a problem and that’s the way I’ve gotten most used to it.”*

Having time to formulate responses without feeling rushed is noted in this response as a benefit of messaging services. Further, some participants primarily engaged in this mode of communication with friends, making them familiar with this way of engaging. However, some respondents preferred having conversations with practitioners over a voice or video call.

*“Maybe zoom calls with services could help, some people might not feel comfortable actually going somewhere to get the help, or maybe they just need to ease into the process.”*

While they still perceived benefit from engaging with clinicians in the same way they would in person, our participants suggested that doing this through digital technology in an environment that is comfortable to them would be beneficial.

The participants reported a desire for voice, text, and video conferences. Some more advanced technological ways of connecting digitally were proposed in the responses. Discord, an instant messaging social platform with a focus on gaming, was proposed as the environment where patient-clinician interactions could take place:

*“I would suggest like a Discord server situation that works like a hotline… I dunno, would have been nice if I could have dropped into something like that when I was 15 and struggling with mental health.”*

Peer interactions with shared experience were also noted as something the participants would like as part of online help-seeking. By connecting with one another, it was noted, individuals would be able to receive support from their online network.

*“Maybe like online meetings with other people that are also living kind of the same situation that myself and talk to each other to have a chance to bring out what is concerning us.”*

This participant suggests the potential benefits of online meetings with individuals facing similar situations as a means of discussing mental health related concerns. They express a desire for a safe and supportive space where they can share their experiences and feelings with others who can relate to their struggles. This highlights the potential value of readily available real-time peer support through communities such as gaming.

### 3.4. Anonymity and Confidentiality

Prominent amongst the responses was the explicit desire for online interactions to consider the importance of information sharing and anonymity. Many participants noted that anonymity, as well as being in an online format, facilitates comfort and results in a successful interaction.

*“pure confidentiality”*

*“Secrecy is important to patients, that would be great”*

*“Confidential options”*

*“More confidentiality. If my information was a little more private I’d feel a lot more comfortable.”*

*“Clear outline of what and what isn’t non confidential”*

*“They should be absolutely and completely anonymous to everyone other than you and the doctor. It should be a feature that there is a way to get treatment without anyone knowing”*

Participants acknowledged a need for more mental healthcare to be underscored by a respect for confidentiality around their personal information, which would make them more comfortable with using a service. They highlighted the importance of having a clear outline of what is and is not confidential during a consultation to ensure that individuals can trust that personal information will be protected. This underscores the importance of maintaining privacy and confidentiality in mental health care to build trust and ensure that individuals feel safe accessing the care they need.

Some participants justified why they believed anonymity was important.

*“Guarantee confidentiality would be beneficial to people like me who worry about what other people might think.”*

*“I also think their [sic] is the fear of someone over hear you on the phone talking.”*

Being perceived by others as needing help for their mental health was uncomfortable for this participant. Hence, anonymity and confidentiality were important factors when deciding on and accessing mental health support. This is particularly important given that participants perceived real consequences if anonymity and confidentiality were not respected:

*“not being able to keep my job if I tell my employer that I am seeking treatment for mental health.”*

*“[I need] job protections to prevent being fired for [accessing mental health services]”*

Participants expressed a need for job protections to prevent being fired for accessing mental health services. They feared that disclosing their need for treatment could have an impact on their employment, highlighting the stigma and discrimination that individuals with mental health concerns may face in the workplace.

## 4. Discussion

This article identified how mental health services can be improved to encourage male videogame players to access them. Research on the help-seeking behavior of videogamers has shown that individuals with a higher risk of mental ill-health are less likely to access help [25]. Given this, this study identified what access factors would facilitate increased access to mental health services for this group. The delivery and features of online services, the practice of service delivery, and anonymity were discussed as important factors relating to access.

The literature on young males and accessing service helps echo the perspectives of the participants in this study. There are a host of areas for improvement in regard to mental health services, including reducing stigma [28] and knowing where to find services [29]. Clearly, increasing the number of men accessing mental health support is a complex challenge. Given that men, in particular young men, face unique challenges when seeking mental health support, services should be readily available and easy to use according to their preferences and skills. The results of this study found that digital online mental health services appealed to videogame players as an avenue of getting appropriate support for mental ill-health. Services should be developed with the consideration that anonymity and confidentiality are highly valued by this group.

Anonymity and confidentiality were identified by our participants as crucial components of accessing mental healthcare. Confidentiality ensures that the personal information shared by patients during treatment remains private and protected. This has been shown to foster trust between patients and healthcare providers [30,31]. Anonymity allows patients to seek mental healthcare without fear of judgment or discrimination, promoting the development of a safe and supportive treatment environment. This has also been found to promote an open and non-judgmental environment between clinicians and patients [32]. Anonymity and confidentiality in mental healthcare are identified as important components of service delivery for this group. Consistent with our findings, research on young men more generally notes that often the rationale for anonymous and confidential mental healthcare may be related to stigma [33]. The fear of being judged by peers, family, and mental health professionals may limit their ability to seek appropriate mental health services. Appropriate considerations of these fears should be built into the design and development of future digital mental health solutions aimed at targeting this group. One way that this fear might be mitigated is if young men have access to high-quality information online to start their help-seeking journey. The information, as emphasized by young men, must be relevant and related to their everyday lives [10]. One study emphasized the importance of engaging young men in communities such as sport and music where they already participate [10], and clearly videogames provide an additional community where these interventions could be targeted.

The respondents, almost unanimously, called for increased access to mental health services. Chan’s help-seeking model [34] is useful as a theoretical underpinning of help-seeking behavior. The model posits a constellation approach, demonstrating that individual, task, and situation factors influence accessing help. Social influences and normative beliefs, such as embarrassment and shame associated with men accessing help [28], play a part in how help is attained. The considerations of these influences must be incorporated into services to address this population’s discomfort with help-seeking. Consistent with research on young men more generally [28,29], our results indicate that anonymity and confidentiality are paramount. Digital mental health services targeting this group need to consider the importance of the perspectives of their users when iterating their design. Incorporating these perspectives and preferences ensures that treatment is value-based [35]. The term ‘value-based framework’ refers to horizontal equity, providing equal care for those who need it, and vertical equity, considering different treatment for different needs or preferences [35]. By considering this vertical component of value-based treatment, as this article does, patients can be co-leaders in their care. Taking on this role is widely associated with improved wellbeing outcomes [36,37]. Based on the results of this study, videogame players value easy-to-access, timely, and genuine mental health services. Value-based treatment in this context refers to increasing the capacity of online mental-health services and addressing the importance of anonymity and confidentiality.

### Limitations and Avenues for Future Research

As noted above, the brevity and ambiguity of some responses led to their exclusion from the analysis. Future research could use interviews or focus groups to give researchers the opportunity to clarify these statements and claims by collecting more in-depth qualitative narrative data on these topics. This rich data on the perspectives and opinions of this group would be beneficial to those designing and implementing help-seeking interventions for this group. The study recruited videogamers via a Tweet from a celebrity videogamer. It would be beneficial to identify and understand the perspectives of videogamers who may not be as engaged with the videogaming community. The demographics of the sample were largely representative of videogame players from Western countries (individuals from Europe, North America and Australia made up 86.7% of the sample). Further research is needed for a more comprehensive perspective of male videogame players in other parts of the world. The perspectives reported in this survey were those of the individuals seeking help and did not consider the perspectives of the clinicians providing mental health services. In conjunction with the reflections reported, these perspectives are essential to providing a more comprehensive perspective of effective service delivery. A combination of consumer and clinician perspectives is an important avenue for future study in order to co-produce service delivery models that are achievable and appropriate.

## 5. Conclusions

Asking male videogame players about their perspectives on increasing help-seeking behavior identified a preference for both online and in-person services that are delivered synchronously, one-on-one with an expert practitioner, and readily available in settings that individuals find comfortable. Services designed for this group should also consider including voice and text-based components in their design. Research suggests maximizing patient outcomes requires engaging men early in their illness trajectory and incorporating these perspectives into the design of services [5,38]. Future research should consider focusing on the experiences and ideas of gaming participants who access mental health services in non-Western contexts. Further, using methods such as focus groups and interviews to capture more of the nuances of digital mental health service design and delivery will enable a more in-depth understanding of how these services can be co-produced with consumers and clinicians.

## Data Availability

Not applicable.
